# Research Progress, Trends, and Current State of Development on PEMFC-New Insights from a Bibliometric Analysis and Characteristics of Two Decades of Research Output

**DOI:** 10.3390/membranes12111103

**Published:** 2022-11-04

**Authors:** Ephraim Bonah Agyekum, Jeffrey Dankwa Ampah, Tabbi Wilberforce, Sandylove Afrane, Christabel Nutakor

**Affiliations:** 1 Department of Nuclear and Renewable Energy, Ural Federal University Named after the First President of Russia Boris Yeltsin, 19 Mira Street, 620002 Ekaterinburg, Russia; 2 School of Environmental Science and Engineering, Tianjin University, Tianjin 300072, China; 3 Mechanical Engineering and Design, School of Engineering and Applied Science, Aston University, Aston Triangle, Birmingham B4 7ET, UK; 4 Department of Biochemistry and Forensic Science, C. K. Tedam University of Technology and Applied Sciences, Navrongo P.O. Box 24, Ghana

**Keywords:** proton exchange membrane fuel cell (PEMFC), hydrogen, PEMFC performance and efficiency, gas diffusion layer, bibliometric analysis, durability

## Abstract

The consumption of hydrogen could increase by sixfold in 2050 compared to 2020 levels, reaching about 530 Mt. Against this backdrop, the proton exchange membrane fuel cell (PEMFC) has been a major research area in the field of energy engineering. Several reviews have been provided in the existing corpus of literature on PEMFC, but questions related to their evolutionary nuances and research hotspots remain largely unanswered. To fill this gap, the current review uses bibliometric analysis to analyze PEMFC articles indexed in the Scopus database that were published between 2000–2021. It has been revealed that the research field is growing at an annual average growth rate of 19.35%, with publications from 2016 to 2012 alone making up 46% of the total articles available since 2000. As the two most energy-consuming economies in the world, the contributions made towards the progress of PEMFC research have largely been from China and the US. From the research trend found in this investigation, it is clear that the focus of the researchers in the field has largely been to improve the performance and efficiency of PEMFC and its components, which is evident from dominating keywords or phrases such as ‘oxygen reduction reaction’, ‘electrocatalysis’, ‘proton exchange membrane’, ‘gas diffusion layer’, ‘water management’, ‘polybenzimidazole’, ‘durability’, and ‘bipolar plate’. We anticipate that the provision of the research themes that have emerged in the PEMFC field in the last two decades from the scientific mapping technique will guide existing and prospective researchers in the field going forward.

## 1. Introduction

The world’s growing energy demand, as well as the negative impact of the world’s major source of energy, i.e., fossil fuel, on the environment, has led to global leaders finding other alternatives for our energy needs [1,2]. Additionally, issues such as price fluctuations in the energy market coupled with the finite nature of fossil fuels make it imperative to find sustainable ways of energy generation [3,4,5,6,7]. It should be noted that for sustainable energy, such as solar and wind energy, the intermittent nature limits their large-scale applications, which opens spatial and temporal gaps between the availability of the energy and its consumption by the end-users [8]. The collective efforts of both stakeholders and investigators have led to more environmentally sustainable, reliable, and cost-effective energy sources such as fuel cells (FC) and water electrolysis [9,10]. 

Hydrogen is seen as an emissions-free energy carrier that can play a significant role in the world’s energy generation sector. It has, in recent years, caught the eyes of researchers globally since its energy density can reach a value as high as 120 MJ/kg, which is estimated to be almost five times higher than that of coal [11,12]. The ability to obtain electricity through the electrochemical conversion of hydrogen as a gas can be obtained through the use of FC, and the FC is a device that has drawn the attention of researchers since they were discovered in 1839 by William Grove [13,14]. The attractiveness of FC is expected to reach maturity by 2030 [15]. The FC technology employs electrochemical reactions to transform the chemical energy in a fuel (mostly hydrogen) as well as an oxidant (mostly oxygen) into electricity directly [16]. Interest in FC has also seen a significant jump since 2007 as a result of innovations that have occurred in FC electric vehicles over the years, as well as the large capacity of stationary FC, which is due to the low or 0% levels of GHG emissions (depending on the fuel type) [17], as well as their ability to attain an efficiency yield of up to about 60% [13].

**Figure 1:**
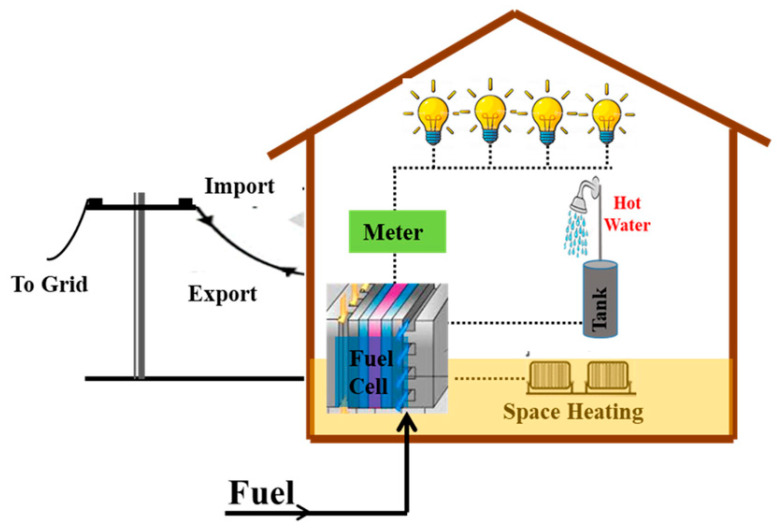
Fuel cell combined heat and power systems [24] (Published under open access).

**Figure 2:**
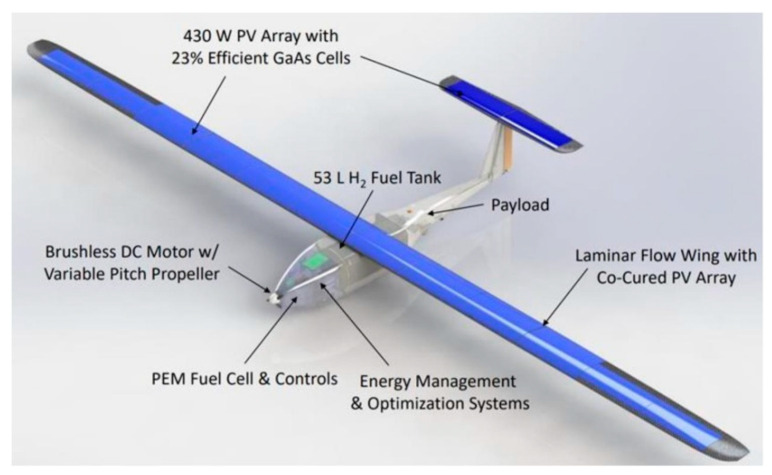
Fuel cell unmanned vehicle [25]. Republished with permission from Elsevier (License no: 5416380516416).

As a result, several alternative technologies have been developed for electricity generation that relies on hydrogen-like Proton Exchange Membrane FC (PEMFC) [18,19,20]. The PEMFC is considered a promising source of power to meet our demand for energy as a result of its high-power density and low operating temperature. Its application is wide, including both the stationary and automotive sectors, as well as the electronic sectors. The application of PEMFC in recent times has evolved from stationary applications for residential purposes, portable and military applications. A fuel cell which combines heat and power systems is presented in Figure 1. In the automotive sector, novel hybrid powertrains are being developed in the quest to reduce the cost of fuel cell electric vehicles (FCEVs). Reports indicate that most automotive manufacturers developed FCEVs, with the likes of Honda, Toyota, and Hyundai among the most prominent ones, with buses and cars being manufactured in pre-series numbers, which are becoming commercially accessible [21]. It is estimated that more than five million FCEVs by 2032 could be sold on the market. In 2020, the number of electric vehicles that were sold was estimated to be around 3 million worldwide [22]. Vehicles that use the PEMFC are more attractive due to their ability to offer the same driving range as that of the combustion engines at the same refueling speed. The level of performance for the PEMFC is very high (about 15 times faster than that of the fast-charge batteries) [22,23]. The development of fuel cells’ combined heat and power systems is one that is projected to reduce the cost of energy utilization in most homes across the globe [24]. Again in 2019, intelligent energy developed a novel 2.4 kW power solution for unmanned aerial vehicles (UAVs). The hybrid tiger unmanned air vehicle developed by the US Naval Research Laboratory is shown in Figure 2.

**Figure 3:**
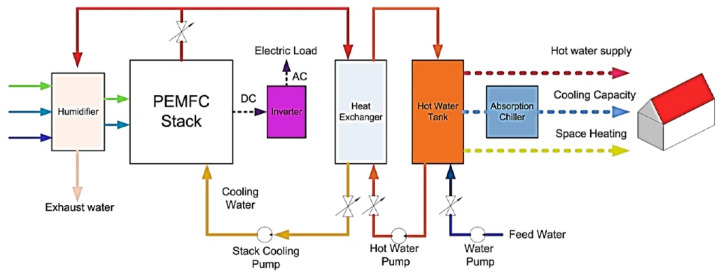
Proton exchange membrane fuel cell absorption chiller [26]. Republished with permission from Elsevier (License no: 5416380834293).

Fuel cells are also being used today for micro-combined cooling, as shown in Figure 3. The system is made up of PEMFC, absorption chiller, tank, and heat exchanger.

**Figure 4:**
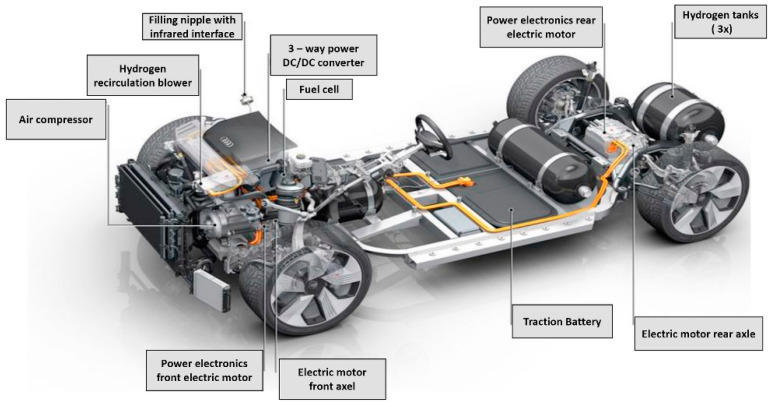
Audi modelled fuel cell electric car [27]. Republished with permission from Elsevier (License no: 5416381285587).

**Figure 5:**
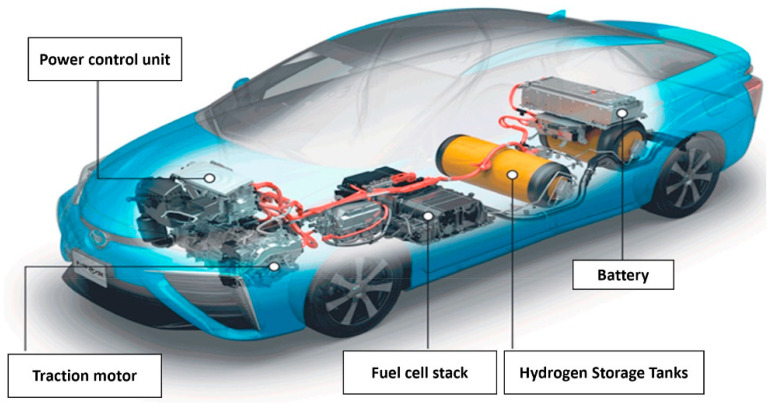
Part of the fuel cell Toyota Mirai [27]. Republished with permission from Elsevier (License no: 5416381285587).

In the automotive industry, there has been significant progress in the redesign of conventional fossil-based powertrains to accommodate motors, fuel cells, and hydrogen tanks, with the ultimate goal of decarbonizing the automotive industry. This has equally resulted in the novel development of powertrains by well-known automotive companies to penetrate the fuel cell market, as shown in Figure 4 and Figure 5.

Despite the significant development in the PEMFC technology, there are some challenges that hinder its use; some of these include performance, cost, and durability, which have to be attended to in order to make it competitive to replace the traditional sources of power [28,29,30,31]. The performance of the PEMFC can be improved by controlling a number of variables, some of which include the control of the air–fuel flow to attain the required temperature and output voltage [32]. This strategy, however, comes with some downsides in relation to risks in the manipulation of the hydrogen, maintenance, and external factors [13].

Several studies have thus looked at how to improve the PEMFC technology to maximize its usage in our daily lives. Khang et al. [16] conducted a study based on Murray’s law to investigate the liquid water transport in a porous layer, as well as a symmetrical biomimetic flow field. They employed the volume of fluid method; a consideration of the dynamic contact angle effects was also done for optimum prediction of the water distribution. Ostroverkh et al. [33] assessed a thin film catalyst with ultra-low metal loading, which ranges between 1 to 200 μg cm^−2^. The preparation was done using a magneton sputtering onto numerous substrates that are carbon-based. The results were composed onto benchmark electrodes, and it indicated that the use of platinum with magneton sputtering could be two orders of degree higher than with the standard Pt/C catalysts while maintaining comparable long-term stability and power efficiency. Ionescu [34] also assessed the impact of geometry of a gas channel by varying the channel heights and widths on a PEMFC’s achieved current density. A uniform variation of the current density was observed along with the FC with channel geometries of 0.8 × 3 mm^2^, 1.2 × 3 mm^2^, and 1.6 × 3 mm^2^ as a result of the large quantity of oxygen that enters the gas flow channel. 

Concerning Pt-based materials’ composition, several studies have been conducted to cut down on the amount of Pt on the catalyst while maintaining high activity and the extension of the robustness of the materials [35]. The use of oxophilic metals, i.e., Ir, Rh, Ru, Ni, Co [36], Os [37], and Sn [38], to alloy Pt to form cocatalysts bi-, tri-, or pluri-metallic has the capacity to enhance its performance and also occasionally lower its price. These alloying elements usually have a strong affinity to the molecules of water, thereby helping the exclusion of CO-species strongly adsorbed over the Pt catalyst surface. These species are further converted into CO_2_ during the course of the small organic molecules’ oxidation [35]. The performance degradation and durability of PEMFC in relation to fuel impurities, air contaminants, and materials that are used to make PEMFC compartments have been shown to have a negative impact on the PEMFC’s performance [39]. In that study, the authors reviewed the impurities, contaminants, and poisoning mechanisms and indicated the importance of enhancing the tolerance of catalyst layers. Song et al. [40] also reviewed research developments of PEMFC in high-altitude environments. According to literature, harsh environmental conditions have a significant impact on PEMFC, and as such, the researchers reviewed the progress of PEMFC that are affected by unconventional conditions that occur as a result of high-altitude climatic environments such as low pressure, vibration, low ambient temperatures, and cathode air starvation. Furthermore, Yang et al. [41] conducted a comprehensive review of PEMFC control strategies by assessing a total of 180 literatures. The control strategies were grouped into nine main categories; it included adaptive control (APC), proportional integral derivative (PID) control, fuzzy logic control (FLC), fault-tolerant control (FTC), observer-based control, robust control, model predictive control (MPC), artificial intelligence control, and optimal control. 

Finally, Yue et al. [42] reviewed the recent progress in the technologies used for the production of hydrogen and their applications in storage and re-electrification. They demonstrated the characteristics of electrolyzers and FC with empirical data as well as the deployments of hydrogen for the storage of energy, co- and tri-generation and transportation were all studied using examples from projects globally. Issues such as efficiency, cost, and durability were identified as the key aspects of the technology that require critical considerations. In [43], the authors reviewed the technical progress of the main components and materials for PEMFC, and the study looked at issues such as the gas diffusion layer, catalyst layer, and bipolar plate. They also considered the development of high-durable processing technologies. 

As demonstrated through the reviewed literature (whether through original or reviewed research) in the above paragraphs, it can be seen that there has been a huge advancement in the area of membrane science in the past few years. However, the problem with such studies, especially the traditional reviewing process, is that they mostly lack a detailed understanding of the research that has taken place in a particular area of study. A bibliometric study thus provides more detail and a fuller understanding of a specific scientific environment or study, which differs from the traditional literature review. The bibliometric way of conducting research allows for an innovative objective view through a reliable and quantitative process. It has also been used as an analytical tool in scientific studies to provide support for researchers with a general idea of various research topics [44]. Bibliometric analysis is a method that is well-established and is employed to assess specific publications in the area of scientific research, as well as trend analysis [44,45,46]. Earlier researchers have conducted bibliometric analysis in various areas such as green economy [47,48], renewable energy [49], alternative fuels in shipping [50], hydrogen energy [51], energy and blockchain [52], green-hydrogen research [53], hydrogen economies [54], and sustainable development [55]. 

Despite the numerous studies on hydrogen energy and PEMFC and their uses, very little information exists on the bibliometric analysis of PEMFC and their recent trends in the energy production space. This study therefore provides a comprehensive assessment of the current research trends and current state of development in the PEMFC sector. This study adopted a clear approach to present the current state of research in the area of PEMFC, and thus, it is expected to provide the research community with an idea about the current happenings in the sector both quantitatively and qualitatively. It could also help shape future research directions both for researchers and other policymakers in the PEMFC space.

The study is presented in six sections, and the current status of the hydrogen economy and trade globally is presented in Section 2. Section 3 presents an overview of hydrogen fuel cells, the method used for the study is presented in Section 4, the results and discussions are in Section 5, and the conclusion is presented in Section 6.

## 2. Current Status of Hydrogen Economy and Trade Globally

A rapid growth in the world’s hydrogen economy could contribute significantly to its geopolitical and geo-economic shifts, which could result in new interdependencies, according to studies from IRENA. The geography of energy trade is changing across the globe, which is an indication of the advent of new centers of geo-political influence built on the generation and utilization of hydrogen as the trading of gas and traditional oil declines [56]. Hydrogen has gained interest globally in recent times as a result of two main reasons. Firstly, the interest of governments around the world has increased with respect to the target of net zero emissions by the middle of this century [57]. In order to realize the 2015 IPCC targets in Paris [58], hydrogen is seen as a key alternative for the reduction of GHG emissions. Secondly, the continuous plummeting cost of electrolyzers and renewables has, in recent times, improved the economic attractiveness and viability of “green hydrogen”, i.e., the production of hydrogen through water electrolysis that is powered by renewable energy. Therefore, green hydrogen can help balance and extend the ongoing revolution in RE [19,57,59]. 

**Figure 6:**
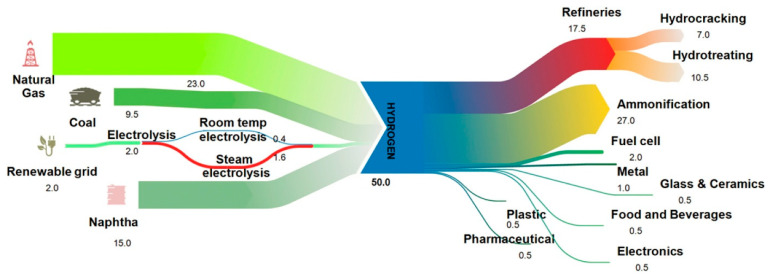
The average hydrogen supply and demand globally, measured in million metric tons [60]. Republished with permission from Elsevier (License no: 5398680782211).

Hydrogen production is mainly from natural gas and oil (Naphtha), which is about 76–77%; production from coal represents about 19–20%, while RE sources take only 3–4% [60]. The hydrogen economy currently mainly serves the chemical and petroleum sectors. It is dominantly used in the elimination of sulfur from “sour” crude oil, ammonia production for agricultural activities, and the cracking of heavy hydrocarbons [19,60,61], as illustrated in Figure 6. 

**Figure 7:**
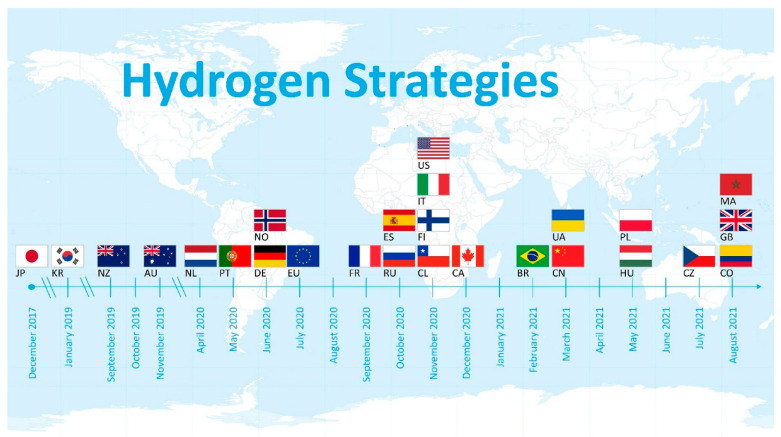
Summary of dates released by various countries’ roadmaps on hydrogen from December 2017 and September 2021 [64] (Published under open access).

Trade in hydrogen across borders is expected to increase significantly, with more than 30 countries and regions already planning for active trade. Countries that are expected to be importers have already commenced the deployment of dedicated hydrogen diplomacy, such as Germany and Japan. Broader economic transition strategies are, however, needed, since hydrogen will not recompense for the losses that will arise from the revenues from the oil and gas sector [56]. According to the IRENA reports, countries such as Morocco, Chile, and Namibia are currently net energy importers and are also set to become green hydrogen exporters. Looking at the huge potentials of regions such as Africa, the Middle East, the Americas, and Oceania could reduce the risk of export concentration; several countries would, however, require technology transfers, investments, and infrastructure at scale [56]. The High Level Group on Hydrogen and FC Technologies of the European Commission, for instance, in 2003 proposed that by 2050 the European Union should attain a hydrogen-based economy and anticipate that about 35% of newly manufactured automobiles by 2040 would be powered by zero-carbon hydrogen [62,63]. A study by [64] published a list of published hydrogen strategies from December 2017 to September 2021, as well as the published draft strategies by countries such as Italy, Poland, Ukraine, Colombia, and Brazil, which could be changed after the consultation phase with the public. The analyzed reports and their dates of publication are presented in Figure 7. It is clear from the reports that a new political strategy was published virtually every month in the last 1.5 years.

## 3. Hydrogen Fuel Cell

**Figure 8:**
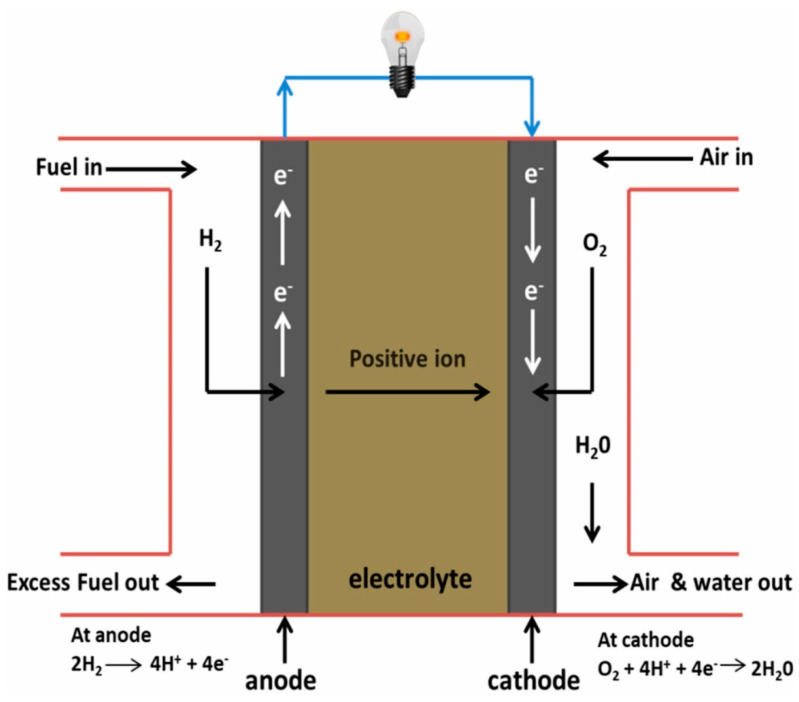
FC general diagram [68]. Republished with permission from Elsevier (License no: 5398681399569) 3.1. Types of Fuel Cells.

There has been a considerable rise in research that focuses on FC, especially due to the revolutionizing ways in which energy is generated. The fuel that is used by the FC is hydrogen. The FC is an electric cell that is different from other cells; it can be fed with fuel continuously so that the electric power it generates can be indefinitely maintained [65,66]. Different types of FC systems exist, and their principle of operation is, however, similar. Three pillars are needed in FCs; these are the cathode, anode, and an electrolyte. The categorization of FCs is done using the type of electrolyte material that was used in its manufacturing. An FC could consist of several individual cells, however, each of them has the three same fundamental parts. The electrolyte can be found between the anode and the cathode [67]. A general representation of FCs is shown in Figure 8. 

There are mainly four families of FCs, and they differ depending on their operating temperature and electrolyte: Proton Exchange Membrane Fuel Cell (PEMFC), alkaline cells (Phosphoric Acid Fuel Cell (PAFC) and Alkaline Fuel Cell (AFC)), Molten Carbonate Fuel Cell (MCFC), and Solid Oxide Fuel Cell (SOFC) [69,70,71]. FCs that operate below a temperature of 200 °C use precious metals (platinum) as catalysts at the two electrodes. MCFC and SOFC, which are high-temperature FCs, can be powered via hydrogen-rich fuels (ethanol, natural gas), special alloys, and tolerate medium-purity hydrogen (99.5%) [69,71,72]. The electrical efficiency of the FC can reach 50% and even 90% depending on the type of FC and power, and if heat is recovered, as the reactions at the cell’s core are exothermic. Currently, most studies are focused on SOFC and PEMFC, whose onboard or stationary applications can supply a number of markets (cogeneration, transport, etc.) [71,73]. As a result, the sections below will dive deeper into PEMFC cells and their current research trends globally.

### 3.1. Proton Exchange Membrane (PEMFC)

**Figure 9:**
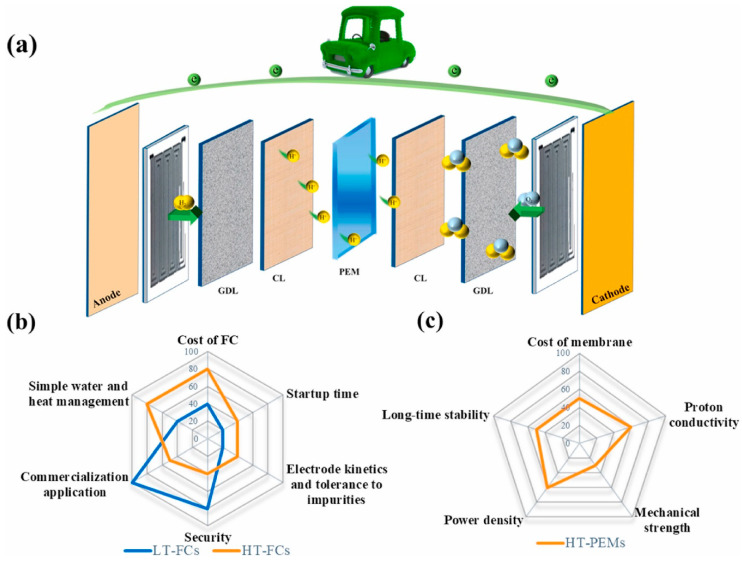
(**a**) A general structure of a PEMFC, (**b**) comparison of LT-PEMFCs and HT-PEMFCs, (**c**) property spider charts of HT-PEMs [79]. Republished with permission from Elsevier (License no: 5398690259427).

One of the major components of the PEMFC is the proton exchange membrane, which was first to the Gemini spacecraft via a polystyrene sulfonic acid membrane in 1960 [74]. Membranes that are desired are those with properties such as decreased fuel and oxidant permeability, compatibility with different fuels, excellent water retention properties, biodegradable, better ion conductivity, facile synthesis, thermal and electrochemical stability, as well as practical mechanical properties to grow in thin structural morphology [75]. The lower cost of fabrication and long life span, while maintaining the properties mentioned earlier, are all key elements of an effective PEM. The main function of a PEM is the transportation of cations and the resistance of anions across electrodes, and this is because functional groups such as SO^3−^, PO^3−^, and COO^−^, which are negatively charged, are present in the side chain of polymers used in synthesizing PEMs to deliver better anion rejection and proton transport across the electrode assembly [68,76]. The PEMFC is basically made up of bipolar plates with channels machined, a membrane electrode assembly (MEA) comprising of PEMs, porous gas diffusion layers, and the catalyst layers [77,78]. A general operating principle of a PEMFC is presented in Figure 9a. PEMFCs can be grouped based on their operating temperature, i.e., high temperature (greater than 120 °C) PEMFCs (HT-PEMFCs) and low temperature (less than 80 °C) PEMFCs (LT-PEMFCs). Figure 9b shows a spider plot that summarizes the difference between LT-PEMFCs and HT-PEMFCs. The HT-PEMFCs, unlike the LT-PEMFCs, are energy devices that are clean with the advantages of reducing CO poisoning, increasing oxygen reduction reaction kinetics, and streamlining water/heat management and cooling systems [79].

#### Operating Principle of the PEMFC

The PEMFC’s basic principle of operation is that there is a flow of hydrogen through the anode feeding channels which passes through a diffusion layer, which reaches the catalytic layer. It is then oxidized into protons and electrons as expressed in the expressions below [19,80]:

(1) $$2H_{2}\to 4H^{+}+4e^{-}$$

The electrons that are released are directed through the catalytic metal as well as the anode catalytic layer’s granulated coal, and finally, it gets to the cathode. Transportation of the protons through the membrane to the catalytic layer of the cathode occurs. Next, the injection of oxygen into the feeding channels of the cathode takes place, which moves through the diffusion layer in the direction of the catalytic layer. At this stage, it reacts with the electrons and protons to produce water, as shown in the relation below:

(2) $$O_{2}+4H^{+}+4e^{-}\to 2H_{2}O$$

The final reaction is therefore written as:

(3) $$2H_{2}+O_{2}\to 2H_{2}O$$

An exothermic reaction takes place at the cathode; the heat that is released depends on the voltage, and this is directly connected to the efficiency of the PEMFC. The reaction is indicated in Figure 3. 

The PEMFC performance largely depends on its temperature; therefore, there is the need to regulate the temperature in a reasonable range to maintain an even temperature distribution of the entire stack to help reduce the consumption of fuel [41]. Equation (4) can be used to assess the energy balance over the electrolyzer system [41].

(4) $$\frac{dT_{el}}{dt}=\frac{NV_{el}I_{el}}{C_{t}}\left(1-\frac{V_{tn}}{V_{el}}\right)-\frac{\left(T_{el}-298\right)}{C_{th}R_{th}}-\frac{m_{cw}C_{wh}\left(298-T_{el}\right)}{C_{th}}$$

where the temperature of the electrolyzer is denoted by $T_{el}$, $C_{th}$ denotes the overall thermal capacity, $R_{th}$ is the overall thermal resistance, $C_{wh}$ is the cooling water-specific heat, $m_{cw}$ is the cooling water mass flow rate, $V_{el}$ is the voltage of single cell, and $V_{tn}$ is the voltage of the thermoneutral cell.

**Figure 10:**
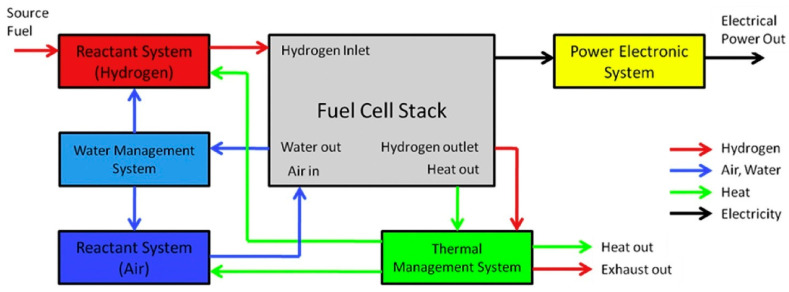
PEMFC schematic diagram [83]. Republished with permission from Elsevier (License no: 5398690664019).

The PEMFC system’s first control strategy is the effective improvement of the electrochemical reactions in the stack through the setting or manipulation of suitable operating parameters such as flow rate of hydrogen and humidity, air, the deterrence of fuel starvation and stack temperature [81,82,83], and the improvement of the durability of the stack through the purging of the anode at the start-up, hence the avoidance of the incidence of high start-up voltage which could lead to the destruction of the stack of the PEMFC. The second control strategy is about ensuring a continuous operation at a specific system output voltage, even when there is a drop in the stack voltage when more current is drawn as a result of polarization through a supercapacitor or a battery, hence improving the performance of the PEMFC system [83,84]. The PEMFC system can be generally classified into at least four subsystems, which are presented in Figure 10 [83]:

- Reaction subsystem.
- Thermal subsystem.
- Water management subsystem.
- Power electronics subsystem.

To be able to enhance the dimensional stability, mechanical strength under dehydration/hydration conditions, and the capacity for water retention, a hybrid organic–inorganic composite membrane was prepared with silica particles spread in a sPPOmatrix. The composite membranes combine the inorganic particle’s stability with the ionic behavior and flexibility of the organic macromolecules, while at the same time exhibiting an enhanced mechanical and thermal stability as well as an improved water retention [85,86]. The particles of the inorganic silica were made in situ using the sol–gel approach, which gives a good dispersion insight of the polymer matrix. This matrix, unlike other similar synthesis by [86,87,88,89], is a simple technique which does not involve organic linker, hence the membrane has a structure that is simple which includes only silica (SiO_2_) and silicon $SiO{\left(CH_{2}-CH2_{-2}-H\right)}_{n\;Replace\;with:\;\left(SiO_{X}\left(OC2H5\right)4-x\right)n}$ particles that are distributed uniformly in the polymer matrix [86].

### 3.2. Cost and Cost Projections of PEMFC

**Figure 11:**
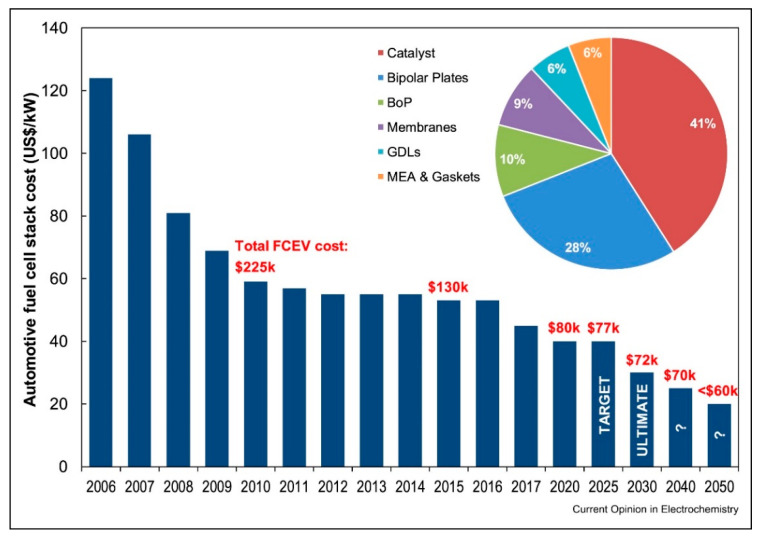
Evolution of automotive FC cost and projections. The PEMFC stack cost is represented by bars, the cost of the total FCEV for some years [91]. Republished with permission from Elsevier (License no: 5398690911349).

Cost and durability are the major challenges that are still facing the use of PEMFC currently. The US Department of Energy’s targets in 2020 for FC systems are US$40/kW with a peak power efficiency of 65% and with 12.5 g of Pt, depending on some 500,000 automobile FC systems per year. The breakdown of the cost for the PEMFC stack found that the catalyst significantly contributes about 41% of the total cost when compared to the membrane, bipolar plate, electrodes and gaskets, gas diffusion layer and the balance of plant costs as shown in Figure 11 [90,91,92]. This is associated with the cost of processing materials and the profit of the manufacturer; these make specialized Pt catalysts more costly than the Pt metal that is untreated. To be able to maintain the competitiveness of the FC in the long term, it is important to work towards a further reduction of the cost to an ultimate of about $30/kWnet, and this will represent a reduction of some 1.35$/kW for the membrane cost in high-volume markets [79]. 

## 4. Methodology for the Bibliometric Analyses

As one of the most comprehensive methods for quantifying scientific output, bibliometric analysis is an effective tool for assessing the contributions and advances made in a particular research field [93]. In this context, the tool is being employed to track the output, impact, structure, and development in the PEMFC research field through the use of various tools and software to analyze the publications in the field. Next, we describe the strategies adopted to identify and map the knowledge domain related to the PEMFC research field in order to present a review that meets the objectives of the present study.

In the current work, the scientometric analysis was conducted based on the following strategies: (i) objectives and research questions are pre-defined; (ii) relevant database(s) is/are selected and used for finding relevant documents based on suitable and comprehensive search terms and phrases; (iii) refine the initial search to reveal and extract documents that are consistent to study goals; (iv) perform analysis based on pre-defined research objectives and questions [94,95].

### 4.1. Bibliometric Analysis Framework

**Figure 12:**
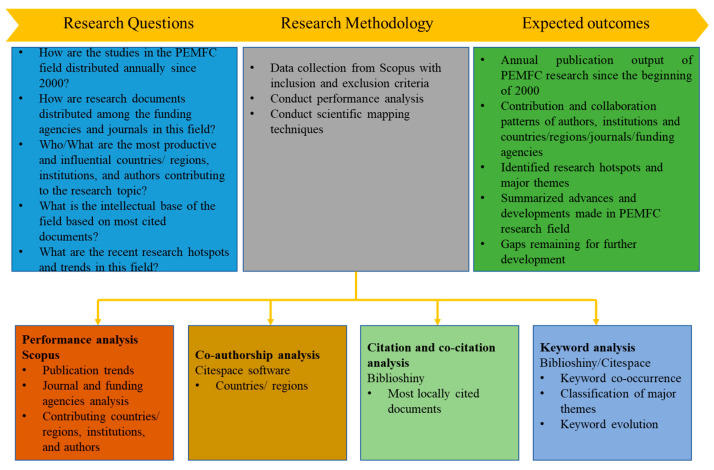
Conceptual framework of the present study.

Performance analysis and science mapping are conventionally the two main types of analyses performing bibliographic data [94,96,97]. In the former, progress has been made in a particular field at the country/region, institution, and author level [98]. We thus conduct this type of analysis to reveal the top-performing countries/regions, institutions, and authors in the PEMFC research field in the last two decades. In addition, the most active journals and funding agencies in the field are presented. The second type of bibliographic data analysis (i.e., science mapping), on the other hand, is useful in ascertaining the relationship between the various actors or components of research [99]. Some analysis performed in the science mapping approach includes co-authorship (author/institution/country), citation analysis, co-citation analysis, and keyword co-occurrence [49]. Figure 12 summarizes the research design of the current study.

### 4.2. Data Sources and Collection Methods

Several databases are available for conducting bibliometric analysis—however, the Scopus database is the world’s largest abstract and citation database for peer-reviewed research [100]. The Scopus database was selected ahead of other popular databases, such as Clarivate’s Web of Science. On the 13th of September 2022, the Scopus database was adopted to search for peer-reviewed documents on PEMFC research. The search query was performed across the article title, abstract, and keyword field as follows: “proton exchange membrane fuel cell*” OR “proton-exchange membrane fuel cell*” OR “polymer electrolyte membrane fuel cell*” OR PEMFC. As the year 2022 was incomplete at the time of investigation, the search spans from 2000 to 2021. The initial search revealed 31,588 documents, of which close to 70% were research articles. As such, the documents were limited to only articles as a way of retrieving original and novel research themes, hotspots, and frontiers on PEMFC. Finally, a sample of 500 documents based on citations was selected to present and summarize the development and trends of PEMFC research since the start of the 21st century.

**Figure 13:**
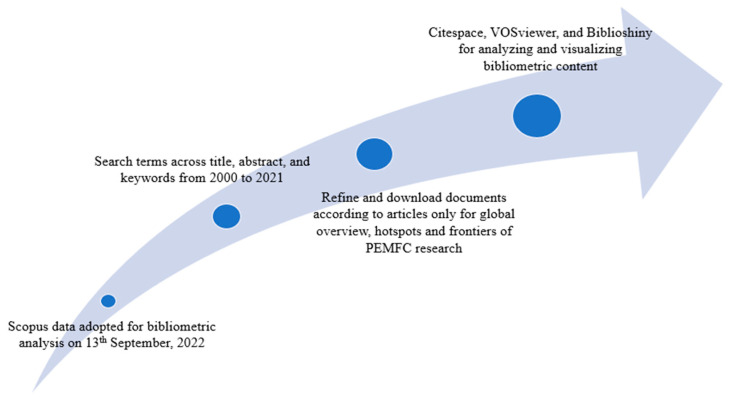
Methodology adopted for retrieving and final dataset on PEMFC research.

To analyze the data, several bibliometric tools were adopted. Scopus has an in-built feature that provides a graphical representation of the annual publication distribution, top-performing countries/regions, authors, affiliations, funding agencies, and document sources of the initial 21,035 documents. Citespace [101], VOSViewer [102], and the bibliometrix package of RStudio (Biblioshiny) [103] were the tools adopted for analyzing and visualizing the bibliometric results of the 500 most cited articles on PEMFC research. Figure 13 summarizes the four main steps taken to reach and analyze the documents retrieved for the present analysis.

## 5. Results

### 5.1. Global Overview of PEMFC Research in the 21st Century

**Figure 14:**
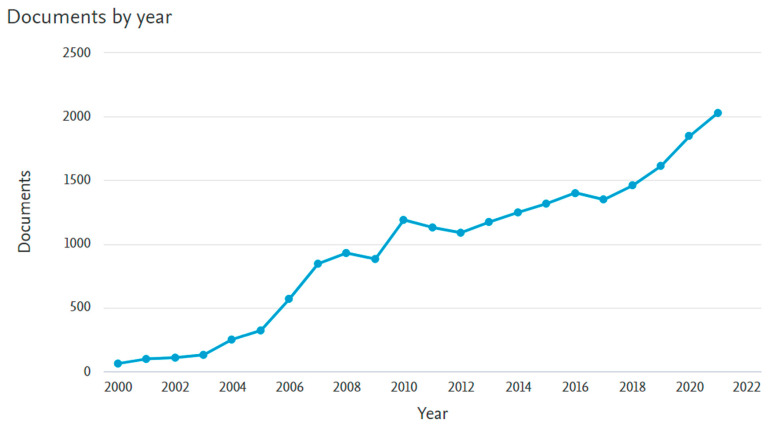
Annual publication output from 2000 to 2021.

In this section, all 21,035 articles available on the subject from 2000–2021 are briefly summarized. The contents of this section include annual publication output, the geographical distribution of publications, research affiliations, key authors, funding agencies, and academic sources (journals). The articles published on PEMFC have seen tremendous growth since the start of the 21st century, with articles averaging an annual growth rate of 19.35%. As seen in Figure 14, the number of articles has increased exponentially in the last five years (2016–2021), amassing a total of 9699 articles, which is only 14% lower than the preceding 16 years combined. The increasing number of publications in the field, especially after 2009, could be attributed to the application of PEMFC being extended from the automotive industry to industrial processes to decarbonize the sector and reduce the carbon footprints of major corporations globally. The development of proton exchange membrane fuel cells in terms of research dates back to the 1950s when it was explored mainly for marine purposes, but this narrative has changed since then, as they are now being used for small-scale stational applications. In 2017, over 490 MW of PEMFC were deployed purposely for the transportation sector. The rest of the units were deployed to the residential market in Japan [104]. The Ene-Farm project has seen more than 200,000 units of PEM fuel cells being sold. This project by the Japanese government, for instance, laid down some primary objectives of providing support for transportation coupled with the residential industry. As part of the program, substantial support was given for research-related activities, the development of prototypes, and subsidies to extend the sale of the product [105]. The development and commercialization of PEM fuel cells in Japan has been largely supported by the government through public funding. The main issue is the fact that the focus is not centered on larger-scale applications. Toshiba has since developed the 100 kW PEMFC. In the United States, the primary purpose for investing in PEMFC was to accelerate the development of Fuel cell electric vehicles. In 2009, the annual R&D budget for PEMFC development was more than USD 80 million, but this dropped from 2009 to 2013 before things became normalized [106]. The development of PEM fuel cells for NASA by General Electric was licensed to Ballard in the 1980s. The company has since championed several research works in the commercialization of PEM fuel cells from an R&D perspective. The focus of Ballard is more inclined toward the transportation industry, but in recent times, the company has explored the application of PEM fuel cells in backup power to 30 kW. The 1 MW ClearGen^TM^ project by Ballard, commissioned at the Toyota Motor Sales in the USA, California, provided power for the sales department. This is one major project that equally revolutionized the PEM fuel cell industry in terms of research [107]. Another project aimed at the development of a PEM fuel cell is the FCH JU CLEARgenDemo, which is a 1 MW PEMFC system. Other companies like ClearEdge [108] also carried out some studies in 2014 and did some experimental work on PEM fuel cells until 2014, when the company decided to focus on phosphoric acid fuel cells. Though the project did not work according to plan, the company managed to raise $136 million for developing PEMFC for commercial and residential applications [109,110]. Companies like Hydrogenics corporation have developed 1 MW fuel cell units with an efficiency lower than 50 percent. Due to their merger with Heijili, a company in the development of fuel cell buses, Hydrogenics corporation is projected by most financial analysts to have a stable future [111]. The South Korean Kolon Water and energy procured three separate units from Hydrogenics corporation. The company signed an agreement with Hydrogenics corporation in terms of sustainable power development in 2014 and has since deployed 1 MW PEMFC units in the Hanwha–Total’s oil refinery in South Korea. Ulsan, in South Korea, procured a 200 kW PEM fuel cell from Horizon Fuel cell Technologies under the Ulsan Technopark (UTP) project. The UTP is a hydrogen town initiative with the ultimate goal of achieving a 1 MW of power obtained via waste hydrogen in the industrial city [111]. The company also did some experiments on the PEMFC they developed for the transportation industry. 

In Europe, financial aid to champion the course of fuel cell development has equally seen an appreciable increase to £58 million pounds from the Framework Program 6 (FP6). Most of these research activities focused more on solid oxide fuel cells and proton exchange membrane fuel cells. Again, in the FP5 (HEAP project), the primary focus of the study was directed toward the UPS market. The goal was to develop a 50 kW PEM fuel cell system. In the FP6, R&D was not studied for applications exceeding 5 kW under the NextGen Cell project. Under the FP7, the ClearGen Demo coupled with the DENCOPEM —2 MW were funded. A spin-out of Akzo Nobel in 1999 led to the development of Nedstack. The 70kW PEM fuel cell stack has supplied more than 2.7 GWh of power continuously for almost seven years [112]. In Antwerp, Solay developed a 1 MW PEM fuel cell generator for one of their plants in 2012. With a 14-million-euro budget supported by the Hydrogen Region Flanders—South Nether program, the project came to fusion. The DEMCOOPEM—2MW project is a PEMFC power plant coupled to a chlor-alkali production unit in Yingkou, China, hence reducing the rate of power use by 20%.

In summary, the appreciable increase in research activities that transcends into publications tends to vary from one country to the other, but there are common underlining factors making us record this high volume of publications in PEMFC. The first is the consistent and remarkable financial support through public funding by various governments globally. Again, with most companies desiring to reduce their carbon footprints as well as meet emission standards for their product or line of business, PEMFC is now projected as the solution to salvage the situation, hence the sudden growth of research interest in this field. Similarly, the versatility associated with the application of PEM fuel cells in global economies has also contributed immensely to its associated research interest globally. Their application in power generation and backup power units has equally widened their interest further in the scientific community. Again, with their cost gradually becoming cheaper in terms of operating and maintenance cost, the research community are investigating the proactive approach to replacing conventional internal combustion engines and diesel engines with fuel cells. 

**Figure 15:**
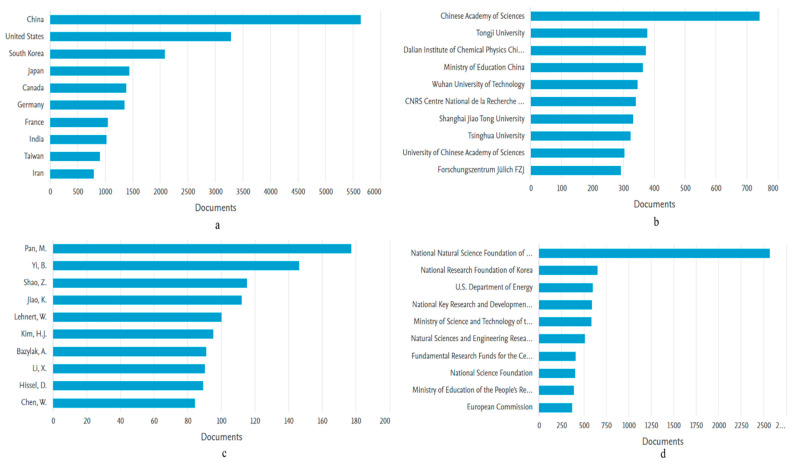
Top performing PEMFC research agents from 2000 to 2021 ((**a**): countries/regions, (**b**): affiliations, (**c**): authors, (**d**): funding agencies).

In Figure 15, the main contributors to the field in terms of article publication are shown according to top countries or regions, authors, affiliations, and funding agencies. China leads the way in PEMFC publications in the 21st century. They have produced 5634 articles, which is 27% of the total number of article publications in the field. Following China are the US (15.6%), South Korea (9.9%), Japan (6.8%), and Canada (6.5%). The dominance of these countries in PEMFC research could be attributed to specific government policies and emissions standard directives aimed at reducing global emissions. To show its commitment to the decarbonization of the global economy, Europe enacted the renewable energy directive in order to sustain the growth of sustainable energy technologies. The focus is to help in the integration of sustainable energy mediums into power-generating companies coupled with a practical utilization of energy storage units for power generation. There is presently more than 16 MW capacity of fuel cells installed for large-scale purposes. An investigation by Roland Berger explored the demand for prime power coupled with combined heat and power in Europe [113]. It was highlighted that there is enormous potential for the large-scale application of fuel cells. There is, for instance, 1.4 GW potential at data centers within the UK. In Germany, they are useful in chemical as well as pharmaceutical companies, water treatment, and the beverage industry. The deployment of fuel cells and CHPs within Europe, Italy, the United Kingdom, and Germany makes them ideal for the gas grid, hence accelerating the commercialization of fuel cells via the utilization of existing infrastructure. Although the potential of fuel cell deployment is positive in most global economies, some researchers argue that the pace has been slower considering the European funding pumped into fuel cells and hydrogen research activities considering FP4 to FP7. In total, nearly 2 billion euros have been committed to these schemes, in addition to other national funding like the NIP program in Germany. Funding for Horizon 2020 for the commercialization of fuel cells at an industrial scale increased to 34 M Euro to date [114]. In Germany, through the National Innovation Program geared towards the integration of fuel cells in the automotive sector, the application of the energy storage device has widened to other sectors of the economy in the last couple of years. Today, the program is being incorporated into maritime-related projects and residential purposes. With a very reliable supply of grid power and lower demand for UPS purposes, businesses in Europe are quite stable. The price of energy tends to vary from one country to the other. Spain has a huge variation between the cost of electricity and natural gas, in excess of 230 EUR/MWH. In Finland, producing electricity from fuel cells using natural gas will be a challenge due to the variation in cost (4 EUR/MWh) in 2017. Penetration of gas grids in these countries is an underlying factor for why lots of investments are being provided for research and demonstration activities relating to fuel cells. Unsurprisingly, as China emerged as the main contributor to the development of PEMFC research, the institutions with the highest performance are mainly Chinese-based. As it stands, only Centre National de la Recherche Scientifique (France) and Forschungszentrum Jülich (Germany) are non-Chinese-based affiliations in the top 10 contributors in the PEMFC field. It is interesting to see that despite the US ranking second according to the top-performing countries, none of their institutions appeared in the top 10 performing institutions. This could be attributed to the fact that most of the publications from the US are relatively distributed evenly among many institutions in the country, thereby reducing concentration on one or few institutions. 

With 0.84% of total publications, the most prolific author in the field is Mu Pan, affiliated with the Wuhan University of Technology (China). This researcher has vast experience in fuel cell research, and according to Scopus citations, the article “A degradation study of Nafion proton exchange membrane of PEM fuel cells” is the most cited article of this researcher. The only non-Chinese-based researchers in the top 10 list are W. Lehnert (Germany), A. Bazylak (Canada), and D. Hissel (France). The trend emphasizes the dominance of China, its affiliations, and researchers in the PEMFC field. The dominance of China could partly be attributed to funding available for PEMFC research. As seen in Figure 9, the National Natural Science Foundation of China (NSFC) is the leading funding agency in the PEMFC research field, and the organization has supported 2564 articles, representing 12% of the total number of articles on PEMFC in the 21st century. This is a significant gap considering that the second top funder (National Research Foundation of Korea) has supported 3% of the total publications, which is only a quarter of what has been funded by NSFC.

**Figure 16:**
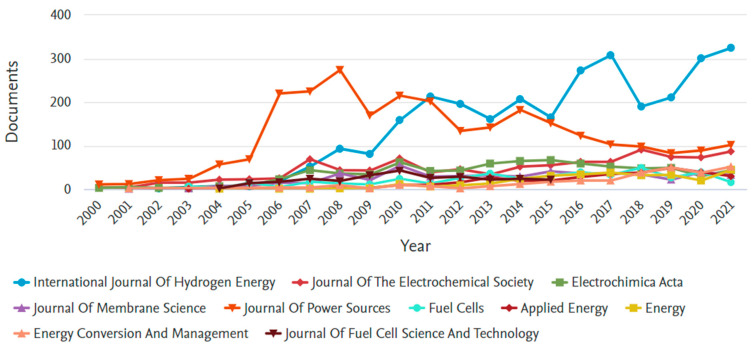
Top performing sources for PEMFC research from 2000 to 2021.

Finally, the top 10 sources for the 21,035 articles are presented in Figure 16. As it stands, the International Journal of Hydrogen Energy leads the way with 14% of the total publications, followed closely by the Journal of Power Sources (12.9%) and the Journal of Electrochemical Society (4.8%).

### 5.2. Research Characteristics, Hotspots, and Frontiers

In this section, the 500 most cited articles in PEMFC research from 2000 to 2021 have been collected and analysed. The contents in this section show the most performing countries/regions and their academic partnerships, most influential articles, research hotspots, frontiers, and evolution.

**Figure 17:**
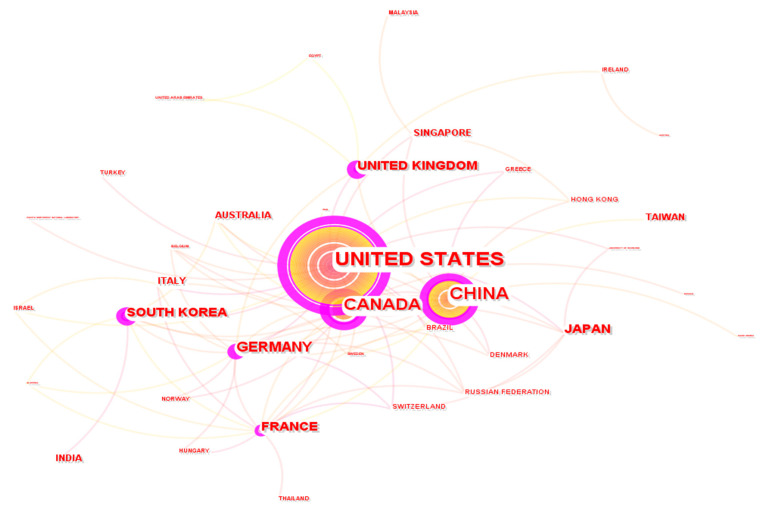
Top performing countries/regions and their collaboration characteristics based on 500 most cited PEMFC articles.

As seen in Figure 17, the main contributors to the publication of the 500 most influential PEMFC articles are USA (210), China (101), Canada (68), Germany (41), and France (31). Because some of the documents are multiple-country publications, those documents are counted multiple times for each country, and as such, the sum of all documents for each country in the list could exceed 500. The trend is quite similar to that of the global trend of the total 21,035 documents, except for the fact that the US and China have switched positions, whereas Japan and South Korea rank below the top 5 spots, giving room to Germany and France. The trend suggests that although Asian countries produce a lot of articles, their publications receive relatively lower attention in terms of citations as compared to that of North America and Europe. This observation is quite consistent with that made in the work of Mao et al. [115]. Collaborations are a means of improving research quality and output, often in the form of knowledge and resource sharing. In fact, the most cited article, “Improved Oxygen Reduction Activity on Pt_3_Ni(111) via Increased Surface Site Availability”, is a collaborative effort between researchers from US and UK-based institutions. Out of the 500 most cited articles, China has collaborated with 13 other countries/regions (collaboration direction from China), with the most collaboration being with Canada (7 times). In the same vein, the US has partnered with 18 countries/regions (collaboration direction from the US), with the most collaboration being with China (22 times). It is of note that research from Africa, Oceania, and South America is heavily underrepresented. The countries in these regions do not have enormous research support as seen in other developed countries, and this could be the main factor for their poor contribution to the development of the PEMFC field. It is recommended that the leading countries/regions with significant funding extend their collaborations to some of these funding-deprived countries in order to drive the global PEMFC field forward.

**Figure A1:**
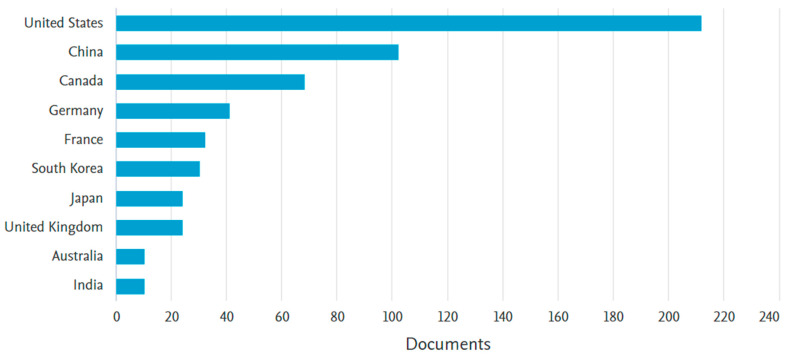
Most productive countries according to the 500 most cited documents in the PEMFC research field.

Figure 15a shows the most productive countries/regions in the field according to total publications. In Figure A1, we show the productivity of the various countries/regions that have contributed to the 500 most cited articles in the PEMFC research field. This is a way of judging country/region productivity by both publications and influence. The 500 documents are, in other words, the most influential documents in the research field under study. It can be seen that the trend in both figures is the same, i.e., most of the countries that have contributed the most to the total document output in the field are also responsible for contributing to the most influential documents. The obvious differences are the changes in spots; for example, the US ranks first for the most influential studies but second for total output, switching places with China in both instances. The same is seen for countries such as Germany, France, South Korea, Japan, and India. It is important to stress that countries such as the United Kingdom and Australia were outside the top 15 countries in the overall document output but found themselves as the main contributors of the most influential documents in the field, swapping places with the likes of Iran and Taiwan. Overall, it can be speculated that the most contributing countries/regions in the field are also responsible for the most influential and quality papers in the field.

**Figure 18:**
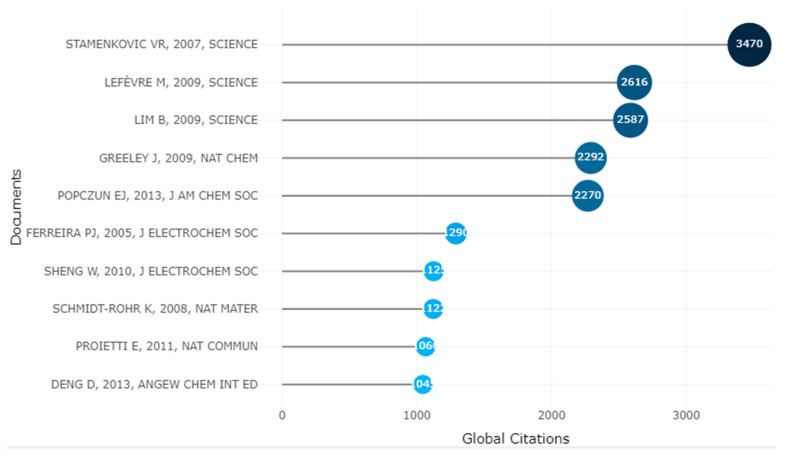
Top 10 most cited PEMFC articles.

Figure 18 shows the top 10 most cited articles on PEMFC research from 2000 to 2021. These articles have arguably formed the basis upon which several studies on PEMFC have been developed in the 21st century. It can be seen that these articles were published more than a decade ago, and this is to be expected, as it takes time for relatively new articles to attract attention from the scientific community through citations. As a matter of fact, the first article (“Single Atomic Iron Catalysts for Oxygen Reduction in Acidic Media: Particle Size Control and Thermal Activation”) published within the last five years (2017) has so far amassed 874 citations and ranks 16th in the all-time list (500 most cited). In this section, we briefly review these top 10 articles by highlighting their objectives and/or key findings. Subsequently, a general perspective is given on all ten articles to find the common link and focus on understanding why they have received such attention within the scientific space and how they have helped shape the PEMFC field. In Table A1, the digital object identifier (DOI) of all ten articles has been provided for quick reference by readers.

Ranked in first place with 3470 citations, the article by Stamenkovic et al. [116] demonstrated an approach to address the slow rate of oxygen reduction reaction (ORR) in PEMFC, which was, at the time of their investigation, the main setback of applying PEMFCs in the automotive industry. They showed that using Pt_3_Ni(111) surface for PEMFC as opposed to Pt(111) surface (Pt_3_Ni(111) is 10-fold more active) and Pt/C catalysts (Pt_3_Ni(111) is 90-fold more active). 

The work of Lefèvre et al. [117] ranks second, with 2616 citations. This article shows improved oxygen reduction activity in PEMFC using iron-based catalysts. At the time of their investigations, the researchers argued that iron-based catalysts for ORR have been poorly competitive against Pt catalysts in PEMFC. As such, the investigators developed and produced microporous carbon-supported iron-based catalysts. The characteristics of this design saw the development of an iron-based electro-catalyst whose current density of a cathode was on a similar level as that of a Pt-based cathode with a loading of 0.4 mg of Pt/square centimeter at a cell voltage of ≥0.9 V.

To improve the catalytic properties and increase the surface activity of Pt catalysts for ORR in PEMFC, Lim et al. [118] controlled the morphology of Pt nanostructures by synthesizing Pd-Pt bimetallic nano-dendrites with excellent characteristics. The Pd-Pt nano-dendrites were 2.5 times more active for the ORR than the Pt/C catalyst on an equivalent Pt basis and five times more active than the first generation supportless Pt-black catalyst. Just like the works of Stamenkovic et al. [116] and Lefèvre et al. [117], this article has also been published in Science and has been cited 2587 times.

Greeley et al. [119] have their article ranking fourth on the list, with 2292 citations. In their study, they consider alloys of Pt and early transition metals for improving the ORR in PEMFC. The design includes ORR electro-catalysts involving Pd or Pt alloyed with scandium (Sc) and yttrium (Y). Results from their study show that the activity of polycrystalline Pt 3 Sc and Pt 3Y electrodes is improved by a factor of 1.5–1.8 and 6–10, respectively, in the range of 0.9–0.87 V as opposed to pure Pt.

The potential for nanoparticle investigations in electrocatalytic activity for hydrogen evolution has also been reported. This study has gained 2270 citations and ranks as the fifth most cited publication. Hollow catalytically active Ni_2_P nanoparticles were used. The materials were faceted with the primary focus of exposing a higher density in terms of the Ni_2_P surface. The incorporation of the nanoparticles presented the best hydrogen evolution reaction activity compared to all other non-noble materials. Hydrogen gas was produced during the process, having a quantitative faradaic yield [120].

With 1290 citations, the study by Ferreira et al. [121] is ranked sixth due to its high impact on the fuel cell research community. The investigation delved into the effect of increasing applied potential (0.9–1.1 V) on equilibrium concentrations from platinum species in Pt/C electro-catalyst samples in 0.5 sulphuric acid at a temperature of 80 °C. It was also deduced that operating proton exchange membrane fuel cells at 0.95 OCV led to a higher platinum surface area loss in terms of a short stack compared to when the cell was operated at approximately 0.75 V. It, therefore, highlights that forming soluble platinum species had a direct correlation with the surface loss in the PEMFC. Using x-ray diffraction and transmission electron microscopy (TEM), a study into the modalities involved in terms of platinum loss at the cathode of the cell was explored further. Analysing the Pt/c catalyst coupled with the cross-sectional membrane electrode assembly using the data gathered from TEM concluded that the process of coarsening of platinum particles happens through distinct approaches. At the nanometre scale, first there is Ostwald ripening, and this causes platinum particle coarsening from 3–6 nm. There is also migration of soluble platinum species at the micrometre scale within the ionomer stage. This phenomenon results in a chemical decline of the species because of hydrogen gas crossover coupled with precipitation of the platinum within the ionomer on the cathodic electrode. This phenomenon causes a complete reduction in the weight of the platinum of the carbon. The approach led to a 50 percent platinum area loss.

The study by Wenchao et al. [122] has garnered 1125 citations since its publication, making it the seventh most highly cited publication. The research work evaluates the phenomena of hydrogen oxidation reaction coupled with hydrogen evolution reaction using polycrystalline platinum, as well as higher surface area supported platinum nanoparticles in 0.1 M KOH with the aid of a rotating disk electrode experimental setup. The study involved fitting kinetic current densities, with the Butler Volmer equation having a transfer coefficient of 0.5. This process was carried out prior to the initial correcting of non-compensated solution resistance using an ac impedance spectroscopy. There were some limitations in terms of the diffusion of hydrogen, especially in the study of the HOR/HER rates on the platinum electrode in a 0.1 M HCIO_4_. Simulating the anode performance in terms of the specific exchange current densities at a temperature of 80 °C showed that despite the ORR cell voltage loss on the cathodic electrode, slower HOR could lead to significant potential anode losses. 

The study by Klaus and Qiang [123] has equally garnered 1122 citation in nature material at the time of writing this publication. The investigation looked into the structure of the Nafion ionomer and utilized a novel algorithm to quantitatively simulate small angle scattering data for hydrated Nafion from previous studies. They noted long ionomer peaks which originated from water channels packed together in the presence of partially hydrophilic side branches, hence leading to the formation of inverted micelle cylinders. The water channel diameters were between 1.8–3.5 nm at 20 vol% water, and the noted average was around 2.4 nm. Nafion crystallites determine the characteristics of Nafion films mechanically due to the formation of physical crosslinks. The crystallites undergo elongation. It must be noted that the water channels and the elongated crystallites are parallel. It was further deduced that there was no correlation between other models like Gierke’s cluster and the scattered data. The newly developed model by Klaus and Qiang [123] is capable of explaining the characteristics of Nafion, i.e., fast diffusion of water as well as hydroxonium ions via Nafion.

Proietti et al. [124] carried out a study on the possible replacement of platinum-based electro-catalysts in proton exchange membrane fuel cells, with the ultimate goal of reducing cost and also extending its application in various sectors. Despite iron-based cathode catalysts being a suitable alternative to platinum, there are issues in relation to their power density compared to platinum-based cathodes because of low mass transport characteristics. A zeolitic imidazolate framework served as a microporous host, and phenanthroline and ferrous acetate formed a catalyst precursor. A 0.75 Wcm^−2^ was attained at 0.6 V, and this was higher compared to the conventional platinum-based cathode. 

Deng et al. [125] also argued in their study that non-precious metal cathode catalysts in PEMFC had issues relating to lower stability in an acidic environment because of leaching. Their study further highlighted the limitations of a catalyst synthesized out of high surface area carbon, polyaniline, and iron coupled with cobalt at elevated temperatures, despite the improved performance when used as an oxygen reduction reaction in PEM fuel cells. The study further investigated the feasibility of designing active and stable non-precious metal as oxygen reduction reaction catalysts. An iron nanoparticle was encapsulated in a compartment via one-step synthesisation at 350 °C with the aid of ferrocene and sodium azide being a precursor. Therefore, the study presented a novel method in terms of electro and heterogeneous catalysts for harsher environments.

A vivid explanation of the accelerated research activities in this field coupled with its high citation can be attributed to the sluggish kinetics of oxygen reduction reaction at the cathodic electrode, which has been a major obstacle in the commercialization of PEMFC due to its lower performance. This has led to the desire for a novel, highly active electro-catalyst for the oxygen reduction reaction in order to meet performance targets laid down by the US Department of Energy and other regulatory organizations worldwide. Despite the successes achieved so far, there is still more room for improvement in terms of evaluating the shape of platinum-based ORR catalysts geometrically and how they affect the properties and performance of proton exchange membrane fuel cells.

**Figure 19:**
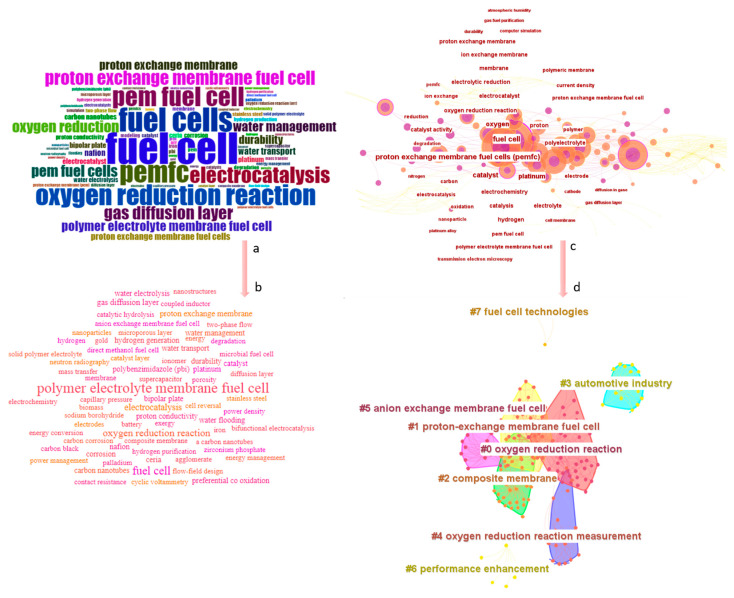
Main research hotspots and frontiers/clusters according to topmost keywords based on the 500 most cited PEMFC articles. (**a**) most used key words (**b**) re-classified key words (**c**) most key words used (**d**) eight main frontiers.

The research hotspots and frontiers/clusters in PEMFC research in the 21st century are shown in Figure 19. Figure 19a comprises the 80 most frequently used author keywords in the 500 most cited articles on PEMFC. Because some terms are synonymous, the words were re-classified by grouping synonymous terms together to generate Figure 19b. According to the results, the top 10 most frequently used keywords are polymer electrolyte membrane fuel cell, fuel cell, oxygen reduction reaction, electrocatalysis, proton exchange membrane, gas diffusion layer, water management, polybenzimidazole, durability, and bipolar plate, with frequencies 117, 70, 50, 32, 21, 17, 13, 12, 11, 9, respectively. The gas diffusion layer serves as a pathway for the flow of the reactive substances from the inlet channels of the anode and cathode electrodes to the catalyst layers where the electrochemical reactions occur. This means that for better PEMFC performance, the reactants should be able to flow freely with limited obstruction in order to reduce pressure drops which will eventually increase the pumping power hence reducing the overall cell performance. It therefore buttresses the justification for accelerated investigations in terms of research in this field. The performance of the gas diffusion layer will eventually affect the water and thermal management characteristics of the cell. Effective water management will increase protonic conductivity and support the easy evolution of hydroxonium ions hence reducing activation polarization, ohmic losses, and mass concentration losses. Ploybenzimidazole membranes are the latest novel types of membranes to allow the operation of lower-temperature PEM fuel cells at elevated temperatures beyond 80 °C. It therefore implies that beyond 80 °C, where water starvation of the membrane would have occurred under normal circumstances, these new types of membranes can ensure the cell still performs at its maximum potential, even at 100 °C. It implies that lower temperature PEMFC can now be used in a harsher environment beyond the usual 80 °C. The gas diffusion layers coupled with the bipolar plates are also well-known components of the fuel cell responsible for providing structural rigidity to the cell. They ensure the fuel is able to sustain any form of pressure being exerted on it through the tightening of the bolts or external pressure being deliberately exerted on the cell. All these keywords highlight the accelerated goal of the fuel cell research community in improving the performance of the PEMFC through material characterization or design optimization.

Figure 19c is similar to Figure 19a but was created with Citespace in order to identify the main research frontier/clusters in PEMFC research. Accordingly, eight main frontiers have been identified, as shown in Figure 19d. We herein discuss the role of these eight themes in PEMFC research in detail.

**#0 Oxygen reduction reaction:** The oxygen reduction reaction defines the overall performance of the fuel cell, as it determines the efficacy of the type of catalyst being used to speed up the electrochemical reactions. In the last decades, different types of catalysts have been explored, largely depending on their cost and how they improve cell performance. Platinum, nanomaterials, etc., are a few that have been explored in recent times.
**#1 Proton exchange membrane fuel cell:** PEMFCs, due to their low operating range, makes them suitable for diverse applications, as explained in previous sessions. The recent spikes globally in power harnessed from the grid have made the research community turn to PEMFCs as suitable replacements for conventional power needed for the automotive industry. It explains the justification for the high number of detailed technical research activities being championed in this field. The other key justification is the fact that PEMFC could be coupled to other energy generation sources for stationary and portable applications. It further explains the recent funding programmes being championed across the globe to further accelerate its commercialization in other global economies. The fact that it is environmentally friendly also means industries will no longer have to struggle to meet emission standards and protocols or pay exorbitant prices for the decarbonization of their business.
**#2 Composite membrane:** Bottlenecks that often come to mind when PEMFC is mentioned in the fuel cell industry is its range of operation. This is because Nafion, being one of the most often used membranes, tends to go through performance challenges beyond 80 °C. Since most industries work with temperatures beyond 80 °C, the search for alternatives to Nafion membranes led to the evolution of composite materials, which are usually made up of Nafion membranes and PTFE fibrous substrates, which became the primary research direction for the fuel cell research community.
**#3 Automotive industry:** To meet emission standards, most automotive companies decided to change their fossil-based powertrains to electric powertrains by replacing the gearbox with an electric motor. This decision received global attention until recently, when questions about the sources of electricity needed for charging the batteries coupled with the duration for charging these batteries became an issue of concern for both the end user and policymakers. This clarion call led to further investigation into replacing the power-generating medium in existing electric vehicles with fuel cells and onboard hydrogen storage units. It, however, led to the accelerated development of fuel cells, particularly for the best interest of the automotive industry. Several research activities aimed at reducing the weight of the cell to meet the standard weight of conventional vehicles have equally been explored but are predominantly geared towards improving the overall performance of the powertrain.
**#4 Oxygen reduction reaction measurement:** The catalyst is what speeds up chemical reactions within the cell, hence its optimization will have a direct impact on the evolution of electrons from the cell, which will reduce the cost of running the cell since hydrogen gas is expensive. This has become a focal research point, largely because there are other materials that could improve the cell performance but are expensive and usually have a detrimental effect on the parameters of the PEMFC.
**#5 Anion exchange membrane fuel cell:** These areas of research are geared toward improving the membrane of fuel cells where anion exchange membranes are used in the separation of the anode and cathode electrodes. This is critical because the membrane serves as a barrier in ensuring the reactants do not mix during the operation of the cell. An effect separation will imply that all electrons being released will be captured, hence reducing the losses within the cell.
**#6 Fuel cell technologies:** There are several types of fuel cell technologies, and each of them has a direct route to the industry or the automotive sector; these technologies are now being explored to augment and reduce carbon emissions from some of these sectors. In some parts of the world, these technologies have equally served as a medium of livelihood to support families as some companies train and teach indigenes on the functionality of the cell and its operation.
**#7 Performance enhancement:** To further accelerate the commercialization of PEMFC, its performance should be improved in terms of the voltage that can be harnessed from the cell, as well as a reduction in the weight of the cell. This explains the efforts being made in designing novel flow plates, membranes, catalysts, etc., to attain the best cell performance.

**Figure 20:**
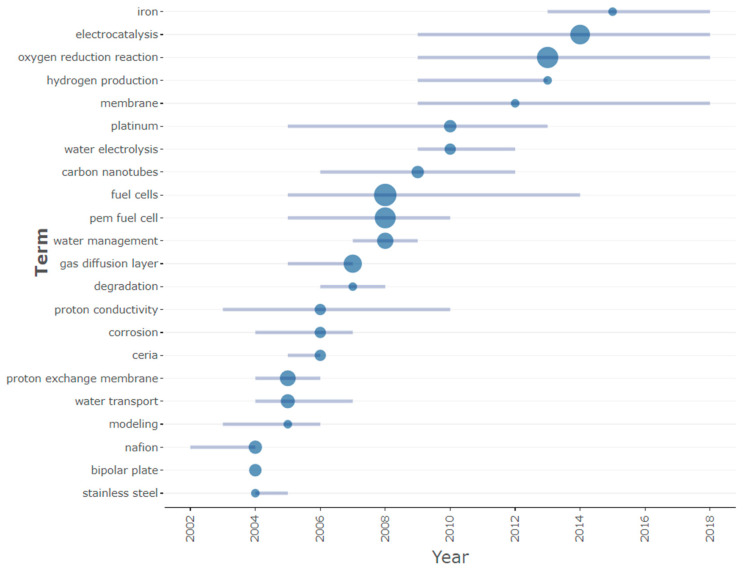
Research topic trend from 2000 to 2021 based on the 500 most cited PEMFC articles.

Figure 20 shows the trending topic for PEMFC research during the study period. From top to down, the trend of terms becomes outdated as new terms gain more attention across the various studies in PEMFC research. Thus, in the last decade, it can be witnessed that attention or focus of studies is related to the PEM fuel cell itself, carbon nanotubes, water electrolysis, platinum, membranes, hydrogen production, ORR, electrocatalysis, and iron. Iron, platinum, and carbon nanotubes are of primary interest in this section—materials with an external dimension of 100 nanometres or less are referred to as nanomaterials. The application of nanomaterials has been utilized in several energy devices like batteries, solar energy, and, lately, fuel cells. The goal of using nanomaterials, especially in PEM fuel cells, is to produce cleaner energy, with a by-product being water and heat. One of the most expensive precious metals in the cell, as explained earlier, platinum, is gradually being replaced with cheaper nano-based materials with higher performance. Some companies are gradually adopting nano-based technology in the development of membranes for PEMFC with higher performance. This approach has allowed the development and evolution of lightweight but longer-life PEMFC. These types of fuel cells will be useful for both industrial-related activities and portable applications like laptops and other smaller gadgets.

**Figure 21:**
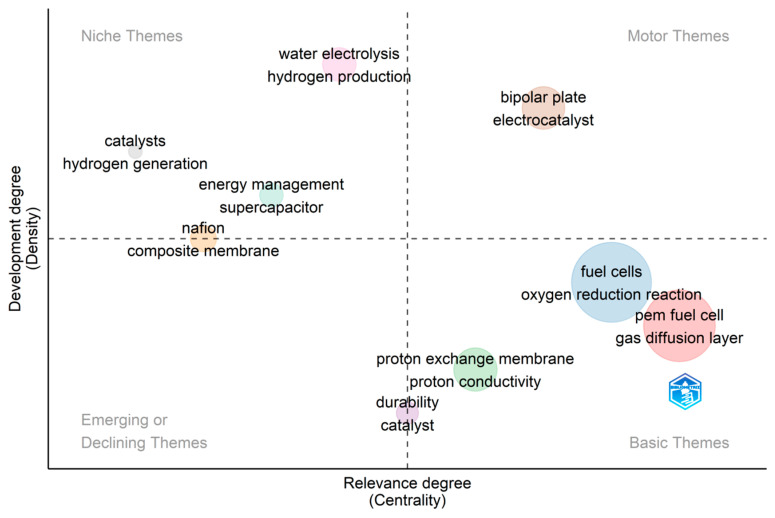
Thematic map based on 500 most cited PEMFC articles.

Figure 21 is a thematic map of PEMFC research based on the 500 most cited articles in the area. This map is useful in identifying core research, peripheral, emerging, and declining themes. The map is divided into four quadrants, and each quadrant explains the role of a theme in an existing research field. Cobo et al. [96] explain these quadrants in brief detail; the first quadrant, also known as the ‘motor themes’, involves themes with high centrality and density. These themes are well-established and developed in the field, and there is little or no room for further development. Bipolar plates, due to their functionality in fuel cells, have been the primary research direction for years. This is because various materials are suitable for the development of flow plates provided; they are light in weight, cheap, have higher mechanical strength, and have good electrical conductivity. With the advancement in materials science, this branch of fuel cell research is equally in the same regard. ‘Niche themes’ are found in the second quadrant, and these themes represent specialized areas of a research field. They are well-developed, but their relevance is only marginal. They rather play peripheral or supporting roles to the main and basic themes for the development of the field. The development of hydrogen fuel cell technology revolves around the availability of good hydrogen infrastructure. The evolutions of various hydrogen technologies give a clear-cut idea of the future of fuel cell technologies. As a research field advances, new ideas are formulated, and old ones become obsolete. These themes are represented in the third quadrant. They contain themes with less development and relevance to the field and represent either declining or emerging themes. The fourth quadrant, known as the ‘basic theme’, involves themes that have high relevance to the development of a field but are relatively less developed than the motor themes. There are still opportunities and areas existing for these themes to undergo further development.

### 5.3. Discussion

The PEMFC has been and continues to be a key research field as the world seeks to find low and zero-carbon solutions to our energy needs. Researchers from different backgrounds in engineering, energy, and environmental science have made significant contributions to the advancement of PEMFC. Despite the fast progress of the field, key questions related to the evolutionary nuances and research hotspots of PEMFC research largely remain unanswered. The existing gap needs to be filled sooner, as having a clear picture of the historical development of an area keeps researchers abreast with the current state of development and reveals which areas will become the main focus of development. The current review contributes to the existing reviews on PEMFC by analysing the research characteristics of the field in the 21st century through bibliometric analysis. The findings reported in this review provide answers to questions such as: (1) how has the PEMFC research field grown over the last two decades? (2) which authors/institutions/countries/regions are the main contributors to the development of the field? (3) which sources (journals) are the key disseminators of the findings made in this field, and (4) what has been the main focus of study in PEMFC research in the last two decades?

It has been established in this study that the PEMFC research field is growing at an annual average growth rate of 19.35%, and the first study in this century was published in 2000 by Ref. [126]. This article talks about a methanol reformer concept, including a reformer, a catalytic burner, a gas cleaning unit, a PEMFC, and an electric motor for use in fuel-cell-powered passenger cars, and their findings have been cited by 62 articles. The last quarter (2016–2021) of the study period has seen the most development in the field, with 46% of the total articles published in this period. The trend shows that the area continues to grow into a major research hotspot in the area of clean fuel technologies. China and the US have been the two main contributors to the development of the PEMFC research field. This is to be expected, as the two countries are the major energy-consuming countries in the world, relying predominantly on fossil-based fuels to meet energy demand. In the US, for example, in 2021, 61% of generated utility-scale electricity was from fossil fuels-coal, natural gas, petroleum, and other gases. China, on the other hand, had coal supplying 55% of the country’s total energy consumption in 2021. These trends in fuel consumption are a threat to the 2015 Paris Climate Agreement and, as such, could partly explain the dedicated efforts of these two world economic giants in finding cleaner alternative sources to power generation, such as the PEMFC. China’s dominance in the field is equally seen at the author, institutional, and funding agency levels, with key actors being Mu Pan, Chinese Academy of Sciences, and National Natural Science Foundation of China, respectively.

Based on the 500 most influential articles on PEMFC research, it has been revealed that the main focus of development in the last two decades has been on improving the oxygen reduction reaction in PEMFC, developing bipolar plates, electro-catalysts, gas diffusion layers, and novel membrane materials. It is evident from these hotspots that the key focus in PEMFC research in the 21st century has primarily been to improve its performance, efficiency, and durability. Some of the catalysts of major interest have included iron, platinum, and carbon nanotubes. Going forward, more novel and promising functional materials with high catalytic activity and stability could become a major research hotspot in the field, which will have an important role in improving the performance of components such as the bipolar plates, which are one of the key components of PEMFC. The economic aspect of PEMFC could also become an area of interest, as reducing PEMFC stack cost could pave the way to achieving higher levels of penetration. Furthermore, thermal and water management of the cell to ensure its applicability in various sectors will accelerate the commercialization of PEMFC. In order to ensure the higher efficiency of the system, waste heat from the cell can be connected to an organic Rankine cycle to generate extra electricity in order to improve the efficiency of the system. Similarly, control logic/strategy to ensure the PEMFC system is able to function properly but judicious use of the fuel connected at the anode will pave the way for the application of the technology in other sectors. The feasibility of cold start of the cell will equally extend its application in the aerospace industry. A proof of concept of a digital twin that can replicate the operation characteristics of the cell in real life will not only reduce cost in terms of research but also ensure industrial partners are able to test the behaviour of a conceptualized system in real-time.

## 6. Conclusions

In this study, a general overview of PEMFC research field from a scientometric point of view has been provided based on 21,035 articles published on the subject since the start of the 21st century. In order to ascertain the evolutionary trends and research hotspots of the field, the 500 most influential articles were selected for further analysis. The key findings of the present review are presented as follows:

- First, the research field is growing at an annual average growth rate of 19.35%, with the first article within the study period published in 2000, which talks about a methanol reformer concept, including a reformer, a catalytic burner, a gas cleaning unit, a PEMFC, and an electric motor for use in fuel-cell-powered passenger cars.
- China and the US have been the major contributors in the field, with a combined 8915 documents. China-based authors, institutions, and funding agencies have been very dominant, which could be attributed partly to the country’s plan to reach a carbon peak by 2030 and carbon neutrality by 2060.
- The International Journal of Hydrogen Energy has been the main knowledge disseminator on a journal level, publishing 14% of the total articles available in the 21 years of the study period.
- Based on the research hotspots identified, the focus of the field in the last two decades has been to enhance the performance, efficiency, and durability of the PEMFC. Keywords such as ORR, electrocatalysis, gas diffusion layer, bipolar plate, water management, and polybenzimidazole have received the most attention from researchers in the field.
- We call on scientists to actively continue developing novel and functional materials with high catalytic activity for improving the overall performance of the fuel cell. The PEMFC stack could be researched in more detail on how to decrease its cost in order to drive the fuel cell’s penetration rate.

Despite the significant findings in this study, our research is not spared from limitations, which are mainly due to database selection (prioritizing Scopus over Web of Science), period of investigation (not including studies before 2000 and after 2021), and limiting research hotspots to the 500 most cited documents. We, however, do not anticipate a major deviation in the trends reported in the current study if the above-mentioned limitations were to be addressed. Future bibliometric analysis could consider other types of fuel cells and compare the trends to our findings to reveal the developmental similarities and differences between the fuel cell types. System modelling and the possible application of fuel cells in the marine industry can also be another active research direction to decarbonize the sector. In the automotive industry, the weight of fuel cells can be reduced by the development of plates and housing units using conductive but lighter materials. Similarly, in terms of reducing the production cost but improving the manufacturing time, the introduction of additive manufacturing should critically be explored in order to achieve these objectives. The flow plate channel geometry design, which is usually machined onto graphite plates, can also be replaced with a more effective design that would improve the water and thermal management of the cell. Further studies into the possible replacement of the platinum catalyst with other metals should be ascertained, and this should be carried out, taking into account the possible reduction of the thickness of the membrane electrode assembly. Alternatively, the flow plate channel geometry design can be replaced with a porous material with good porosity to support the even distribution of the reactive substance within the catalyst layer of the cell. Additionally, the PEM electrolyser’s research evolution and trends could be researched to assess how the field of hydrogen production in PEM electrolyser influences the field of hydrogen consumption in PEMFC. 

## Data Availability

Not applicable.

## Appendix A

**Table A1:** Top 10 most influential studies on PEMFC.

Rank	Paper DOI	Citations
1	10.1126/science.1135941	3470
2	10.1126/science.1170051	2616
3	10.1126/science.1170377	2587
4	10.1038/nchem.367	2292
5	10.1021/ja403440e	2270
6	10.1149/1.2050347	1290
7	10.1149/1.3483106	1125
8	10.1038/nmat2074	1122
9	10.1038/ncomms1427	1066
10	10.1002/anie.201204958	1045
